# A Wafer-Level Stacking Scheme Based on Hybrid Etching and Low-Temperature Bonding for High-Performance MEMS Devices

**DOI:** 10.3390/mi17060651

**Published:** 2026-05-25

**Authors:** Pengfei Li, Xin Yan, Yunjie Yang, Leilei Meng, Xiwen Zhang, Haiyan Wang, Qianbo Lu

**Affiliations:** 1 School of Marine Science and Technology, Northwestern Polytechnical University, Xi’an 710072, China; pfcrazy@mail.nwpu.edu.cn (P.L.);; 2 Flight Automatic Control Research Institute, Aviation Industry Corporation of China, Xi’an 710089, China; 3 State Key Laboratory of Flexible Electronics (LOFE), Institute of Flexible Electronics (IFE), Northwestern Polytechnical University, 127 West Youyi Road, Xi’an 710072, China

**Keywords:** wafer-level stacking, low-temperature curing, MOEMS accelerometer, hybrid etching, wet etching corner compensation

## Abstract

Silicon micromachining serves as the foundational enabling technology for high-precision MEMS inertial sensors. However, the relentless pursuit of enhanced sensitivity and multi-functionality in emerging applications encounters a fundamental bottleneck when confined to two-dimensional scaling. The evolution toward complex three-dimensional (3D) stacking architectures is an inevitable trajectory for devices including MEMS inertial sensors, yet performance is constrained by the limitations of conventional processes in fabricating and integrating intricate 3D hollow structures. Specifically, uniformity in large-area deep silicon etching, structural integrity of convex corners in wet etching, and residual stress induced by multi-layer wafer bonding have emerged as critical, shared challenges. To address these issues, this paper proposes a triple-layer wafer-level stacking scheme that synergistically combines wet/dry hybrid etching with low-temperature adhesive bonding. This stacking scheme incorporates an innovative linear compensation model for wet-etched convex corners, enabling high-precision fabrication of complex corner structures under deep etching conditions. Furthermore, a collaborative strategy involving temporary bonding and plasma flow-field optimization improves the uniformity and integrity of dry etching for large perforated structures. A low-temperature triple-layer wafer-level stacking process is developed, encompassing precise adhesive dispensing, optical alignment, and a stepped low-temperature curing profile, thereby achieving highly symmetric 3D integration with controlled adhesive distribution. The efficacy of this stacking scheme is validated through the fabrication of a symmetrically stacked triple-layer MOEMS accelerometer sensing element. Test results demonstrate a noise floor as low as 0.40 µg/√Hz and a bias instability of 1.81 µg over 10 min. Compared with a double-layer counterpart, improved performance is obtained. The wafer-level stacking scheme established in this work not only provides a viable pathway for pushing the manufacturing limits of high-precision inertial devices but also offers a generic methodology for tackling complex hollow structure formation and low-temperature integration, holding referential value for broader applications in high-precision 3D microsystems.

## 1. Introduction

The core driving force of microelectromechanical systems (MEMSs) technology stems from the continuous evolution of bulk silicon micromachining and wafer-level packaging integration [1]. In particular, bulk micromachining processes represented by deep reactive ion etching (DRIE) and wafer bonding have become the cornerstone for manufacturing inertial sensors such as accelerometers and gyroscopes. The relentless pursuit of higher aspect ratios and more intricate structures has propelled iterative advancements in device performance [2,3]. Nevertheless, as cutting-edge applications like geophysical monitoring, precision navigation, and deep-sea exploration impose stringent sub-µg requirements on sensor noise and stability, existing process routes confront a fundamental dichotomy: to mitigate mechanical–thermal noise and enhance resolution, the proof mass must be increased or the support beam stiffness optimized, inevitably demanding larger chip areas or more complex multi-layer architectures [4]. Concurrently, the prevailing trends of system integration and miniaturization continually compress the physical volume of devices, posing significant challenges to conventional planar processes and monolithic integration schemes.

Achieving a large proof mass through multi-layer wafer stacking is widely recognized as a core technological pathway for realizing sub-µg and even ng-level noise floors [3,5]. Triple-layer or multi-layer symmetrically stacked architectures not only multiply the effective proof mass but also effectively suppress cross-axis sensitivity through structural symmetry [6], thereby reconciling the demands for high performance and compact form factors. However, the engineering realization of such complex 3D stacked structures entails a series of formidable process challenges. In anisotropic wet etching of (100) silicon, severe undercutting at convex corners compromises profile accuracy, and for etch depths exceeding 200 µm, conventional layout-based compensation methods become increasingly inadequate [7,8]. Dry etching presents its own difficulties: when DRIE is applied to layouts combining large perforated areas with narrow beams, the micro-loading effect introduces significant local etch rate variations, while the large exposed silicon area exacerbates within-wafer non-uniformity through reactant depletion, making critical dimensions such as beam width difficult to control [9,10]. Moreover, the presence of large perforated regions can lead to helium leakage from the electrostatic chuck and renders the released fragile structures vulnerable to mechanical or electrostatic damage during subsequent handling [11]. Finally, traditional high-temperature, high-pressure bonding processes tend to introduce substantial thermal mismatch residual stress into brittle silicon microstructures, resulting in warpage or failure that severely degrades long-term stability [12,13]. Collectively, these issues represent critical bottlenecks that must be addressed to advance the performance of MEMS inertial devices.

In response to these common process bottlenecks hindering the development of high-performance MEMS devices, this paper proposes and systematically validates a wafer-level stacking scheme predicated on hybrid etching and low-temperature bonding. First, a linear compensation model for wet-etched convex corners, based on mask edge retreat rate, is established, simplifying a complex two-dimensional compensation problem into a one-dimensional linear calculation and enabling high-precision convex corner formation under deep etching conditions. Second, a collaborative dry etching scheme employing “Halo structures + temporary bonding + edge ring flow-field optimization” improves etching uniformity and process stability for large-perforated structures. Finally, a low-temperature triple-layer wafer-level stacking process is developed, incorporating a stepped low-temperature curing profile and a minimal-pressure strategy to reduce thermo-mechanical loading during integration. Using a high-performance MOEMS accelerometer sensing element as a test vehicle, this paper elaborates on the critical steps and technical principles of the proposed stacking scheme, validates its engineering efficacy through noise and stability test results, and subsequently explores its potential implications for the broader landscape of high-precision micro/nano manufacturing.

## 2. Process Scheme Overview

**Figure 1:**
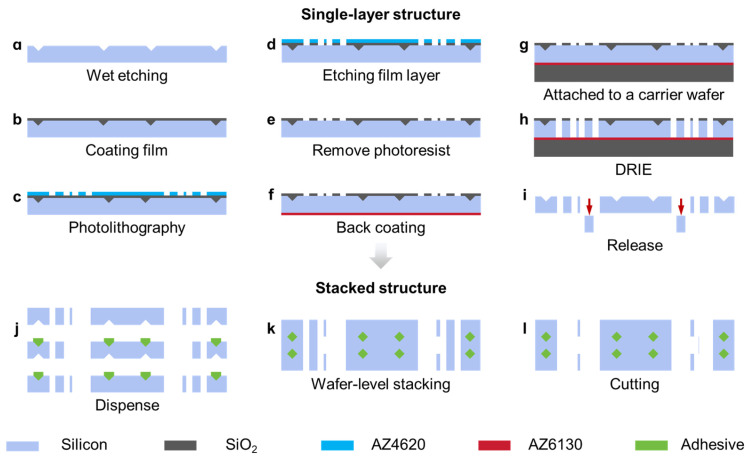
Schematic diagram of the triple-layer wafer-level stacking process flow. Single-layer structure: (**a**) KOH wet etching for adhesive reservoirs and thinning; (**b**) SiO_2_ coating film; (**c**) photolithography; (**d**) etching of film layer; (**e**) AZ4620 photoresist removal; (**f**) AZ6130 back coating; (**g**) attachment to a glass carrier wafer; (**h**) DRIE for structural definition; (**i**) release of structural layer. Stacked structure: (**j**) precise adhesive dispensing; (**k**) wafer-level stacking and curing; (**l**) dicing/cutting into discrete elements.

The proposed triple-layer wafer-level stacking scheme vertically stacks three 4-inch (100 mm-diameter) single-crystal silicon wafers (Okmetic, Vantaa, Finland), each 400 µm thick. A hybrid shaping strategy combining wet etching for targeted thinning and dry etching for release is employed. The specific process flow is schematically illustrated in Figure 1.

Initially, each silicon wafer layer is patterned individually. Taking the middle without-beam layer as an example: first, KOH wet etching is performed to create adhesive reservoirs required for subsequent bonding and to form locally thinned regions (step a). Subsequently, temporary bonding technology is utilized to attach this layer to a carrier glass wafer, providing robust physical support (steps b–g). Next, DRIE is conducted to release the core-suspended beam-proof mass structures (step h). Finally, the temporary bonding layer is removed, yielding a freestanding perforated structural layer (step i). The upper and lower with-beam layers are fabricated using analogous wet/dry hybrid processes. Ultimately, an adhesive is precisely dispensed into the designated reservoir areas on the three layers. Wafer-level stacking assembly and curing are completed using a dedicated triple-layer alignment system, forming a highly symmetric, large-proof-mass 3D stacked sensing element. This stacking scheme ingeniously combines process modules to leverage the full thickness of three wafers for mass multiplication while relying on the symmetric support of the upper and lower layers to ensure overall structural reliability and rigidity.

## 3. Wet/Dry Hybrid Bulk Silicon Micromachining

### 3.1. High-Precision Convex Corner Control in Deep Wet Etching Based on a Linear Compensation Model

**Figure 2:**
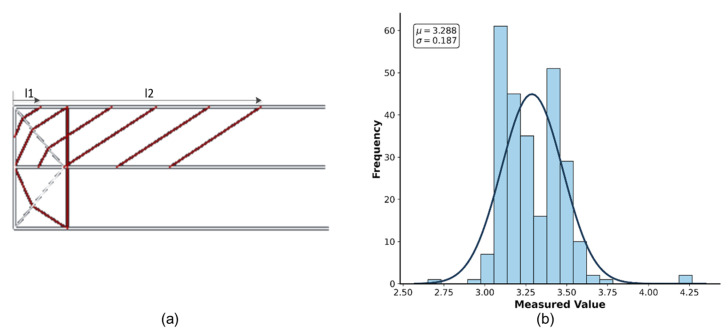
(**a**) Schematic of the convex-corner compensation linear design; (**b**) statistical plot of the recession ratio measured under approximately 35 wt% KOH etching at 80 °C.

Traditional convex corner compensation methods rely on adding compensation patterns to the layout, including square and oblique rectangular structures [14,15,16]. More compact or layout-efficient compensation structures have also been reported for space-limited or adjacent convex corners [17]. In the present design, square and oblique rectangular patterns were selected because the investigated convex-corner regions have sufficient local layout space and because the objective was to calibrate a reproducible linear compensation model under deep etching conditions. However, for deep etching exceeding 200 µm and involving complex structures with numerous irregular convex corners, empirical compensation methods can still require cumbersome process iteration [7,8]. To overcome this limitation, this paper proposes a convex corner compensation model based on the linear accumulation of the mask edge retreat rate. The KOH etchant used for model calibration was prepared by mixing 40 wt% KOH solution with deionized water at a 70:13 ratio, yielding an approximately 35 wt% KOH solution. Etching was conducted at 80 °C, and the measured etching rate was approximately 0.95 µm/min. Under these fixed conditions, the ratio of the lateral etch rate of a mask edge parallel to the <110> crystal direction to the vertical depth etch rate can be treated as a statistically constant value [18]. As determined experimentally and shown in Figure 2, this characteristic ratio has a mean value of 3.288 with a variance of 0.18. This finding allows the complex problem of compensating arbitrarily shaped polygonal convex corners to be reduced from two-dimensional layout design to a one-dimensional linear calculation: the total required compensation linewidth, L, is approximately the product of the target etch depth, D, and this characteristic ratio.

By way of illustration, the total length of a linear edge region is denoted as l. After an etch time t0, the edge recedes laterally by l1; after a further time t1, the recession is l2. The total compensation length L = l1 + l2 can be determined by multiplying the desired etch depth by the factor of 3.288. The specific values of L, l/width, and target etch depth D used in the validation patterns are listed in Table 1. This methodology enables prediction of the morphological evolution of any complex polygon during the etching process, allowing the required compensation to be calculated directly at the design stage without recourse to time-consuming empirical iteration or extensive process trials. As depicted in Figure 2, this method transforms intricate pattern compensation into linear prediction based on rate parameters, reducing process development complexity, particularly for structures containing numerous irregular convex corners as featured in this work. The compensation accuracy can be pre-evaluated based on the statistical precision of the rate measurement, specifically by analyzing the ratio of lateral edge recession to etch depth on beveled surfaces. Moreover, the method exhibits portability; by measuring the rate parameters for specific KOH solution compositions and temperature conditions, it can be applied across different process lines, thereby establishing a foundation for subsequent high-precision stacking and bonding.

### 3.2. Optimization of Deep DRIE for Large-Perforated Structures

Deep reactive ion etching is employed to define the core functional features such as suspended beams and proof masses. The presence of large-area perforated regions adjacent to free-standing beam structures presents formidable process challenges, primarily manifesting as micro-loading effects, degradation of within-wafer uniformity, and the risk of damage to released structures.

**Figure 3:**
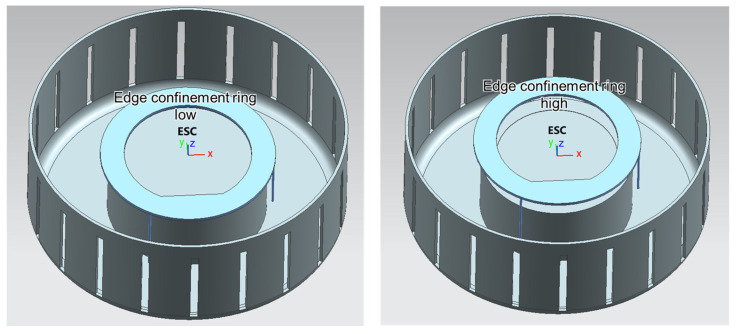
Schematic of edge confinement ring in low (**left**) and high (**right**) positions within the chamber.

To address these challenges, a collaborative etching optimization strategy was implemented:

1. Layout Uniformity Optimization: Dense dummy patterns (Halo structures) were added to large blank areas of the mask layout to balance the exposed silicon area across the entire wafer. This effectively suppressed the micro-loading effect [19], improving cross-wafer etch depth uniformity to within ±3% and controlling sidewall verticality between 89.5° and 90.5°.
2. Flow-Field Uniformity Optimization: To counteract reactant depletion and byproduct accumulation associated with large exposed areas, the process recipe was adjusted to maintain the edge confinement ring in an elevated position within the etch chamber during processing (as illustrated in Figure 3). In this configuration, the ring acts as a flow distribution plate, effectively constraining the plasma flow field and thereby improving within-wafer etch uniformity from ~8.8% to within 3% [20].
3. Temporary Bonding for Process Protection: To mitigate issues of helium leakage and post-etch handling damage associated with perforated structures, a temporary bonding technique was adopted [11,21]. In this work, AZ6130 photoresist (MicroChemicals GmbH, Ulm, Germany) was used as the temporary degradable adhesive. AZ6130 was coated onto the glass carrier support and soft-baked to provide temporary adhesion, after which the silicon device wafer was bonded to the carrier glass wafer before DRIE. After processing, the temporary bonding layer was released by immersing the bonded assembly in acetone until debonding occurred. Residual AZ6130 on the silicon wafer was then removed by high-power oxygen plasma dry-cleaning. This step provides physical isolation between the substrate and the E-chuck, reducing the risks of helium cooling leakage and electrostatic damage. Furthermore, it offers rigid mechanical support for both the fragile released structures and the Halo features, preventing collapse or damage during processing.

**Figure 4:**
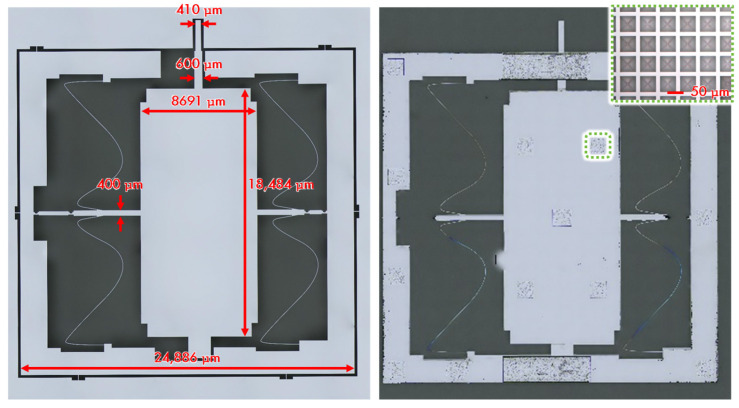
Optical image of the wet-etched adhesive reservoir with measured dimensional annotations.

Through this synergistic “layout–flow field–support” optimization, the process achieves high-yield fabrication of large-scale suspended-beam-proof mass structures (as shown in Figure 4) and extends the capability of standard DRIE to address the processing of extreme microstructures, such as those featuring ultra-high aspect ratios, slender beams, and large-area proof masses.

## 4. Triple-Layer Wafer-Level Low-Temperature Stacking Process

The three discrete silicon structural layers fabricated via the hybrid micromachining process must be precisely integrated into a unified sensing element through stacking and bonding. To reduce the residual-stress concerns commonly associated with traditional high-temperature, high-pressure bonding of large-scale, compliant perforated structures [12,22], a low-temperature wafer-level stacking scheme was developed, leveraging the advantages of both temporary bonding and adhesive joining [21,23] while accounting for the specific structural characteristics.

### 4.1. Precise Adhesive Dispensing and Material Properties

**Figure 5:**
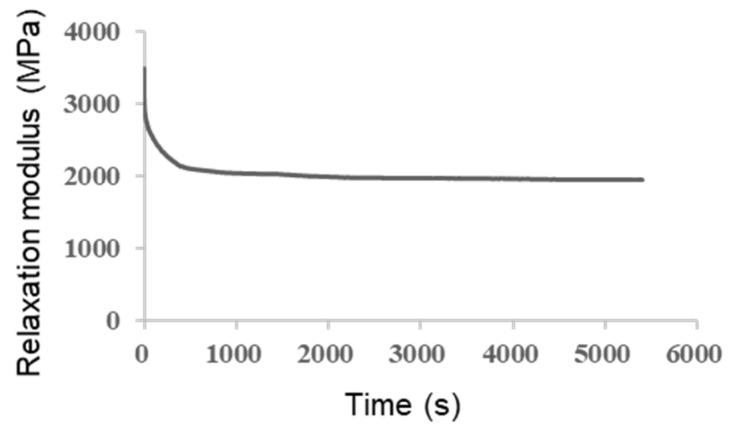
Relaxation modulus of the proprietary adhesive.

Precise deposition and material characteristics of the adhesive are critical for managing deformation and bonding reliability in the stacked assembly [24]. An automated PC350 dispensing system (Musashi Engineering, Inc., Mitaka, Japan) with a positioning accuracy of ±5 µm was utilized to quantitatively deposit a custom-formulated adhesive, Paste18, into the wet-etched reservoirs. Paste18 consists of an epoxy resin matrix, an imidazole-based curing agent, and calcium carbonate filler mixed in a mass ratio of 60:5:20. This adhesive has a curing shrinkage of approximately 0.6% and a coefficient of thermal expansion (CTE) of approximately 25 ppm/K, together with high bond strength. Its relaxation modulus characteristics, shown in Figure 5, help mitigate thermal mismatch stress within the multi-layer stack.

The adhesive bonding strategy is potentially transferable to other wafer-level MEMS integration processes because it relies on local dispensing into predefined adhesive reservoirs and low-temperature curing, rather than on a device-specific structural feature. The same bonding adhesive has been adopted in our previous MOEMS relative gravimeter work published in Nature Communications [5] and in inertial-navigation accelerometer products developed by the Flight Automatic Control Research Institute. Nevertheless, the method is not intended to replace all conventional wafer-bonding approaches. For applications requiring ultra-high-vacuum hermetic sealing, high-temperature post-bond processing, or an electrically conductive bonding interface, additional process optimization or an alternative bonding method may be required.

### 4.2. Flexible Fixturing and Precision Alignment

**Figure 6:**
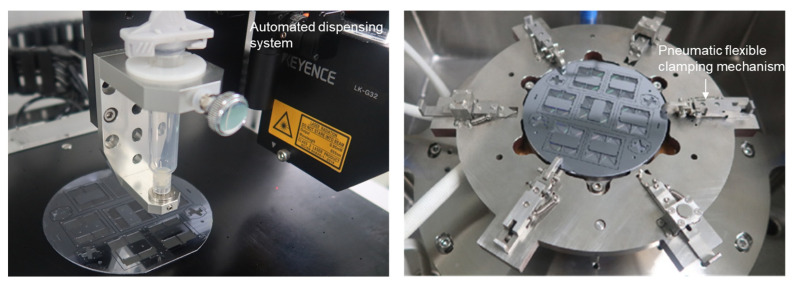
Photograph of the dispensing, alignment and clamping fixture.

To achieve simultaneous precision alignment of three silicon wafers during the stacking process, a dedicated triple-layer alignment and clamping system was customized and jointly developed with Suzhou Meitu Semiconductor Technology Co., Ltd. (Suzhou, China), as depicted in Figure 6. This system integrates mechanical pre-alignment pins with high-resolution optical vision feedback, enabling layer-to-layer alignment accuracy within ±3 µm. Following alignment, a pneumatic flexible clamping mechanism applies a uniform contact pressure ranging from 50 to 200 kPa, ensuring intimate wafer contact while preventing structural micro-deformation attributable to uneven clamping forces.

### 4.3. Stepped Low-Temperature Curing with Minimal-Pressure Strategy

**Figure 7:**
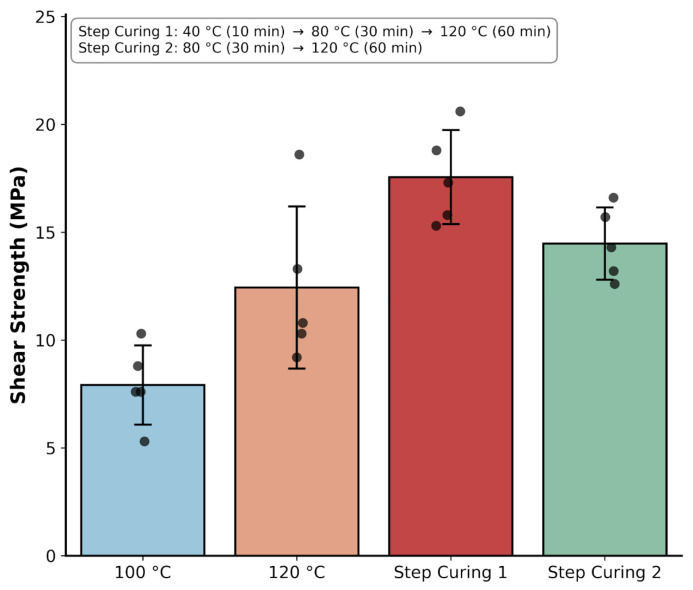
Comparison of shear strength under four curing processes: constant curing at 100 °C for 100 min, constant curing at 120 °C for 100 min, Step Curing 1 (40 °C for 10 min, 80 °C for 30 min, and 120 °C for 60 min), and Step Curing 2 (80 °C for 30 min and 120 °C for 60 min). Bar heights represent the mean shear strength (n = 5 per group), and error bars indicate the standard deviation (SD). Black dots show the individual raw data points.

Conventional high-temperature (>300 °C) and high-pressure (≥1 MPa) bonding processes readily induce uncontrollable thermal and mechanical stresses within brittle silicon microstructures, potentially leading to warpage or failure. Through comparative experimental optimization, four curing protocols were evaluated, including two constant-temperature processes and two step-curing processes. The conditions labelled 100 °C and 120 °C in Figure 7 correspond to constant-temperature curing at 100 °C and 120 °C, respectively, each for 100 min. Step Curing 1 is a three-stage curing process: 40 °C preheating for 10 min to promote adhesive leveling and degassing; 80 °C holding for 30 min to trigger preliminary cross-linking; and 120 °C curing for 60 min to achieve final bonding strength. Step Curing 2 is a two-stage curing process consisting of 80 °C for 30 min followed by 120 °C for 60 min. As shown in Figure 7, specimens cured using Step Curing 1 exhibited an average shear strength of 17.56 MPa at the bond interface, with higher strength and better uniformity than the other curing protocols compared here.

**Figure 8:**
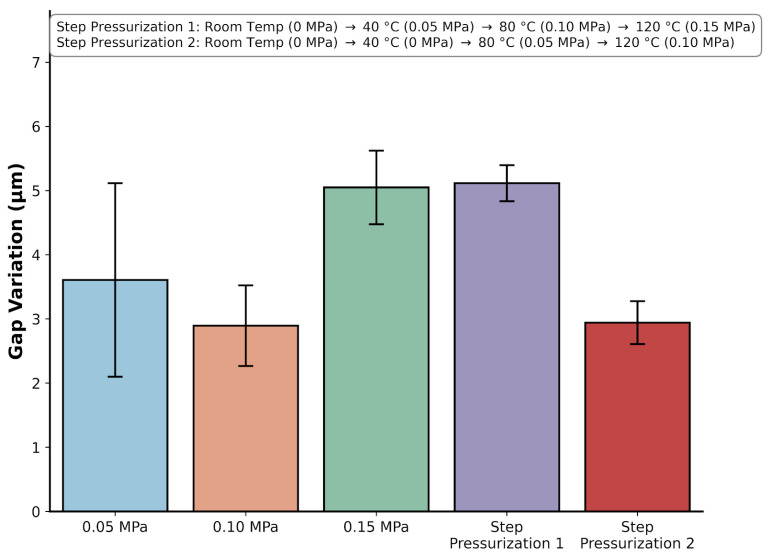
Comparison of bonding-gap standard deviation under different pressurization schemes during the curing process. The gap was measured at four positions across the two bonded interfaces; a smaller standard deviation indicates better pressure-assisted curing uniformity. The constant-pressure groups were maintained at 0.05 MPa, 0.10 MPa, or 0.15 MPa during the complete thermal profile (40 °C for 10 min, 80 °C for 30 min, and 120 °C for 60 min). Step Pressurization 1 used 0.05 MPa, 0.10 MPa, and 0.15 MPa during the 40 °C, 80 °C, and 120 °C stages, respectively; Step Pressurization 2 used 0 MPa, 0.05 MPa, and 0.10 MPa, respectively. Bar heights represent the standard deviation of the measured gap values, and error bars indicate the SD of repeated samples.

Another challenge in device assembly pertains to thermo-mechanical coupling during pressurized curing. To evaluate the uniformity of pressure-assisted curing, the interfacial gap was measured at four positions across the two bonded interfaces, and the standard deviation of these gap values was used as the uniformity metric. A smaller standard deviation indicates better curing/bonding uniformity. As shown in Figure 8, five pressurization schemes were compared. For the first three schemes, constant pressures of 0.05 MPa, 0.10 MPa, or 0.15 MPa were maintained throughout the same thermal profile (40 °C for 10 min, 80 °C for 30 min, and 120 °C for 60 min). Step Pressurization 1 applied 0.05 MPa at 40 °C, 0.10 MPa at 80 °C, and 0.15 MPa at 120 °C. Step Pressurization 2 applied 0 MPa at 40 °C, 0.05 MPa at 80 °C, and 0.10 MPa at 120 °C. In the present comparison, Step Pressurization 2 provided the lowest gap standard deviation, indicating improved bonding uniformity while avoiding excessive mechanical loading in the early curing stage.

### 4.4. Symmetric Process Design and Integration

**Figure 9:**
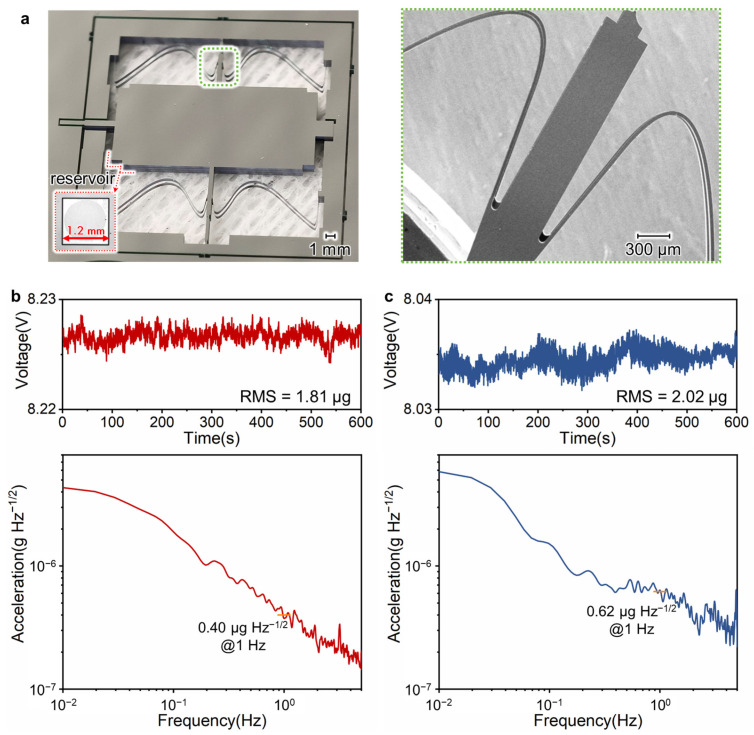
(**a**) Photograph of the symmetrically stacked triple-layer structure with a scale bar and magnified insets showing the adhesive-filled reservoir and the stacked connecting rod; (**b**) ten-minute static output and acceleration power spectral density of the triple-layer MOEMS accelerometer; (**c**) ten-minute static output and acceleration power spectral density of the double-layer reference device.

The structural layout adopts a highly symmetric stacking configuration of “with-beam layer—without-beam layer—with-beam layer,” as illustrated in Figure 9a. The upper and lower with-beam layers are fabricated from the same process batch. Consequently, any trapezoidal cross-sectional errors in the beams introduced by DRIE are mirrored and effectively cancel each other out upon assembly. This ensures that the center of mass of the sensitive element closely coincides with the geometric center of the support beams, inherently suppressing cross-axis sensitivity at the design level. Simultaneously, the wet-etched adhesive reservoirs confine the bonding agent. A magnified image of the adhesive-filled reservoir has been added in Figure 9a, showing that the adhesive remains within the storage groove after bonding. This design reduces the risk of adhesive intrusion into the critical beam-proof mass motion gaps and improves the predictability and consistency of the device’s mechanical behavior.

## 5. Device Validation and Performance Results

The efficacy of the proposed wafer-level stacking scheme was validated through the fabrication of a sensing element for a high-performance MOEMS accelerometer, with performance compared against a double-layer counterpart.

The triple-layer stacked sensing element was integrated with an optical readout module (comprising a light source, a shutter rod, and a differential photodetector) to form a complete MOEMS accelerometer. Static sensitivity testing of the triple-layer device demonstrated excellent linearity (R^2^ > 0.999) and a mechanical sensitivity of 193.91 µm/(m/s^2^).

Long-term static testing was conducted on a vibration isolation platform. Analysis of the acceleration power spectral density, as shown in Figure 9b, reveals that the triple-layer stacked device fabricated using the proposed stacking scheme achieves a noise floor (at 1 Hz) as low as 0.40 µg/√Hz and a 10-min bias instability of 1.81 µg. In contrast, the double-layer reference device exhibited a noise floor of 0.62 µg/√Hz and a bias instability of 2.02 µg, as shown in Figure 9c.

These test results indicate that the triple-layer symmetrically stacked structure realized through the proposed stacking scheme provides improved noise performance and long-term stability compared with the asymmetric double-layer configuration. This performance improvement is attributed to the high degree of symmetry achieved by the stacking scheme, which helps suppress the accumulation of process-induced errors and cross-axis coupling effects.

## 6. Conclusions

This paper has presented and validated a wafer-level stacking scheme based on hybrid etching and low-temperature bonding, addressing the shared challenges of fabricating complex 3D hollow structures and achieving controlled low-temperature integration for high-performance MEMS devices. A highly versatile hybrid etching framework was established through the development of a linear compensation model for wet-etched convex corners and the adoption of a collaborative DRIE strategy encompassing “Halo structures—temporary bonding—flow-field optimization.” This framework improves the capability and uniformity of standard MEMS processes for fabricating large-scale, high-precision perforated silicon structures, remains fully compatible with existing process modules, and is readily scalable to four or more stacked layers. Building upon this foundation, a low-temperature triple-layer wafer-level stacking process was realized by integrating precise adhesive dispensing, high-accuracy alignment, and an optimized stepped low-temperature, minimal-pressure curing profile, thereby achieving highly symmetric, high-strength, low-defect 3D integration while reducing the thermal load and residual stress concerns associated with conventional high-temperature, high-pressure bonding. The stacking scheme was validated through the fabrication of a MOEMS accelerometer sensing element, which exhibited an improved noise floor and bias instability, confirming its capability to provide a reliable manufacturing foundation for next-generation sub-µg and higher-precision inertial instruments. In summary, the wafer-level stacking scheme established in this work not only offers an effective solution for manufacturing core components of high-precision inertial devices but also demonstrates a generic methodology for tackling complex 3D structure formation and low-temperature integration, holding referential significance and engineering value for high-value-added microsystem fields, including optical MEMS, microfluidic chips, and RF devices.

## Figures and Tables

**Table 1:** Comparison of compensation patterns and etching results. D denotes the target etching depth, and L and l/width denote the geometrical parameters used in the mask compensation design.

No.	Description	Layout	Etching Result
1	45° Oblique Rectangle L = 63.293 µm; l/width = 40 µm; target D = 20 µm	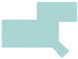 a	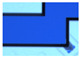 b
2	45° Oblique Rectangle (over-compensated) L = 73.293 µm; l/width = 50 µm; target D = 25 µm	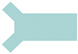 c	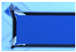 d
3	45° with Right Angle L = 63.293 µm; l/width = 40 µm; target D = 20 µm	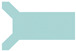 e	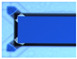 f
4	Square Corner Compensation side length = 40 µm; overlap = 15 µm; target D = 20 µm	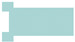 g	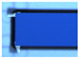 h

## Data Availability

The raw data supporting the conclusions of this article will be made available by the authors on request.
